# The Effect of Radiation on the Immune Response to Cancers

**DOI:** 10.3390/ijms15010927

**Published:** 2014-01-10

**Authors:** Bonggoo Park, Cassian Yee, Kyung-Mi Lee

**Affiliations:** 1 Global Research Laboratory, Department of Biochemistry and Molecular Biology, Korea University College of Medicine, 126-1 Anam-dong 5-ga, Seongbuk-gu, Seoul 136-713, Korea; E-Mail: parkbg40@korea.ac.kr; 2 The University of Texas MD Anderson Cancer Center, 7455 Fannin St, Houston, TX 77054, USA; E-Mail: cyee@mdanderson.org

**Keywords:** radiation, abscopal effect, cell therapy, trafficking, recognition

## Abstract

In cancer patients undergoing radiation therapy, the beneficial effects of radiation can extend beyond direct cytotoxicity to tumor cells. Delivery of localized radiation to tumors often leads to systemic responses at distant sites, a phenomenon known as the abscopal effect which has been attributed to the induction and enhancement of the endogenous anti-tumor innate and adaptive immune response. The mechanisms surrounding the abscopal effect are diverse and include trafficking of lymphocytes into the tumor microenvironment, enhanced tumor recognition and killing via up-regulation of tumor antigens and antigen presenting machinery and, induction of positive immunomodulatory pathways. Here, we discuss potential mechanisms of radiation-induced enhancement of the anti-tumor response through its effect on the host immune system and explore potential combinational immune-based strategies such as adoptive cellular therapy using *ex vivo* expanded NK and T cells as a means of delivering a potent effector population in the context of radiation-enhanced anti-tumor immune environment.

## 1. Introduction

The effect of ionizing radiation on healthy individuals depends on the total dose and dose rate of radiation exposure. High-dose ionizing radiation, given acutely at high-dose rate, is generally considered to be detrimental, causing apoptosis, DNA damage, and transformation of cells into tumor cells [1,2]. Radiation induces stress signals in normal mammalian cells, activating DNA repair pathways and cell cycle checkpoints, resulting in recovery or cell death. The same modality, that is toxic to normal cells, has also been one of the most effective tools for cancer therapy. This DNA damaging property usually gives rise to considerably distinct outcomes depending on the type of cancer, be it a more remediable lymphoid or germ cell cancer or resistant epithelial solid tumor [2].

Radiation therapy mediates tumor cell killing primarily via apoptosis and often leads to necrosis and mitotic catastrophe due to the DNA damage evoked within the tumor microenvironment. In some cases, radiation also induces autophagy and senescence in tumor cells, which contribute to its anti-tumor properties. For example, autophagy, which can be elicited by exposure to the mTOR inhibitor, rapamycin, is a survival pathway in some radiation resistant cancer cells such as glioma or parotid carcinoma cells, but can also paradoxically enhance radiosensitivity in these same cells. This radiation-enhancing effect seems to result from heterochromatin remodeling, irreversible growth arrest, and premature senescence. At the cellular level, enhanced radiosensitivity is likely due to the restoration in tumor suppressor RB1 activity and its subsequent suppression of E2F transcription factor 1 (E2F1) activity [3,4]. For prostate cancer, which is considered relatively radio-resistant, treatment with the phorbol ester, 12-*O*-tetradecanoylphorbol 12-acetate (TPA), can also enhance radiation-induced cytotoxicity. TPA inhibits ATM activity resulting in de-repression of the ceramide synthase pathway and enabling radiation-mediated activation of *de novo* ceramide synthesis, which induces apoptosis [5].

Senescence, a common occurrence in normal cells owing to limits in proliferation, is one of the major pathways leading to tumor growth retardation. Although senescence is generally the result of telomere shortening during cell proliferation, radiation-induced senescence is quite different in that the growth arrest is promoted by the activation of tumor suppressor p53 as well as p21 [6].

Cells of the immune system, like most radiosensitive tumors, can also be rapidly dividing, and are vulnerable to radiation. Radiation exposure induces apoptosis in mature NK cells as well as T and B lymphocytes and lethal damage in bone marrow stem cell precursors of monocytes and granulocytes. In individuals receiving heavy doses of radiation, for example, atomic bomb survivors, both mature lymphocytes and bone marrow stem cells were severely damaged, causing profound depletion of granulocytes and natural killer cells.

However, radiation treatment, under certain conditions can also enhance the immune response. Owing to its cytotoxic effect on tumor cells, radiation exposure can provide a source of antigen that is well-suited for cross presentation by the host antigen-presenting cells (*i.e*., dendritic cell) which in turn can induce an antigen-specific immune response. In addition, other immunopotentiating properties of radiation therapy may be observed by its influence on the tumor microenvironment to enhance cell trafficking to tumor sites, its effect on modulating antigen presentation itself and direct effects on the immune effector cells. An understanding of the biochemical and molecular mechanisms in these distinct pathways evoked by radiation can lead to the development of more efficient radiotherapies with beneficial biological and immunological consequences [6,7]. In the following sections, we will discuss the effect of radiation on the endogenous immune system (Section 2) and then in the context of a transferred immune response as an adjunctive modality to adoptive NK and T cell therapy (Section 3).

## 2. Effects of Radiation on the Endogenous Immune System

### 2.1. Systemic Antitumor Immune Response (Abscopal Effect)

Local radiotherapy on cancer cells occasionally induces the regression of metastatic cancer at distant sites which have not been irradiated apparently through induction of adaptive immune responses. This phenomenon has been called an abscopal effect and can be attributed to the induction and enhancement of endogenous anti-tumor innate and adaptive immune responses. The mechanisms surrounding the abscopal effect are diverse and include trafficking of lymphocytes into the tumor microenvironment, enhanced tumor recognition and killing via up-regulation of tumor antigens and antigen presenting machinery and, induction of positive immunomodulatory pathways.

Cytokines play an important role in the abscopal effect. In one case, a Japanese patient receiving radiotherapy for thoracic vertebral bone metastasis, experienced spontaneous regression of an unrelated hepatocellular carcinoma. Pre- and post-analysis of serum cytokine levels revealed marked elevation of tumor necrosis factor-α following radiotherapy, suggesting that the abscopal related regression may involve such cytokines as part of the host immune response [8]. Another radiation-induced cytokine, IFN-β, has been shown to enhance T cell-dependent tumor regression by increasing the cross-priming capacity of tumor-infiltrating dendritic cells in mouse model, an effect that can be mimicked by delivery of exogenous IFN-β into the tumor tissues without radiation [9]. That this abscopal effect is mediated by immune cells is supported by the observation that exogenous administration of chemokines following local radiation therapy can lead to enhanced killing of tumors at distal sites. This abscopal effect was tumor-type independent, involving infiltration of CD8^+^ and CD4^+^ lymphocytes and NK1.1^+^ NK cells into the tumor sites of mice [10].

For these reasons, the abscopal effect is being intensely studied in the field of immune-based therapies. Cytotoxic T-lymphocyte-associated antigen 4 (CTLA-4), one of the negative regulators of cytotoxic CD8^+^ T cells, has been targeted as a means to activate anti-tumor immune CTLs in mouse xenografts [11]. This effect can be attributed in part to an anti-CTLA4 mAb-mediated decrease in the threshold of activation among endogenous tumor-reactive T cells. In addition, it has recently been shown that local radiotherapy and CTLA-4 blockade significantly reduce the motility of tumor infiltrating lymphocytes at tumor sites, allowing them to engage in stable interactions with tumor targets [12]. The NKG2D ligand, RAE-1 (Retinoic acid early inducible-1), is up-regulated on irradiated neoplastic cells; interaction with its receptor, NKG2D, on cytotoxic T cells leads to costimulation and enhanced tumor cell killing. These signals, transduced by the TCR, NKG2D and CTLA-4, contribute to the stability of the immunological synapse [12]. The association appears to be mediated in part by increases in the antibody responses to multiple tumor antigens after radiotherapy in a melanoma patient [13].

Multiple factors contribute to the development of an abscopal effect and involve the interplay of irradiation and induction of adaptive immune responses leading to elimination of tumor cells at distant sites. These factors will be discussed in the following sections.

### 2.2. Tumor Antigens and Antigen Presentation

#### 2.2.1. Cytotoxic T Lymphocytes and Dendritic Cells

Anti-tumor CTL responses, which represent the outcome of an abscopal effect primed by the irradiated tumor cells, seem to play a significant role in establishing the antigen-specific immunity that accompanies radiation therapy. When immunogenic tumor cells were treated with ionizing radiation in the presence of a DNA repair inhibitor (veliparib), and then injected into tumor-bearing mice, an antitumor CTL response was generated leading to elimination of established tumors [14]. Further evidence that antigen-specific T cells are elicited after radiation therapy is borne by studies demonstrating, significant reduction in systemic tumor burden in CD8^+^ T cell dependent fashion after ablative radiation therapy (RT) to local tumors in mice with high-dose radiation [15]. It is postulated that following RT-mediated tumor cell death, T cell priming likely occurs through dendritic cell (DC) cross presentation of released tumor antigens in draining lymph nodes, leading to rejection of the primary or metastatic tumors.

Antigen presentation by DCs seems to be crucial to RT-induced CD8^+^ T cell dependent anti-tumor immunity in murine models. Antigens can be endogenously processed and loaded onto MHC class I molecules or added exogenously. Radiation seems to differentially affect these two antigen presentation pathways: presentation of endogenous antigen is blocked by, whereas presentation of exogenously pulsed peptide was enhanced in irradiated DCs leading to a favorable anti-tumor T cell responses [16].

#### 2.2.2. MHC Class I and II

Radiation therapy facilitates cell surface expression of MHC class I molecules by one of three mechanisms during radiation in dose-dependent manner. These mechanisms include (1) radiation induced protein unfolding and degradation, resulting in an increased intracellular peptide pool; (2) radiation-induced enhancement of protein synthesis resulting also in an increased intracellular peptide pool; and (3) increased diversity of the intracellular peptide pool due to the generaton of radiation-specific peptide antigens. Accompanied by augmented surface expression of MHC class I molecules, an increase in the quantity and/or diversity of the peptide pool leads to an overall increase in the number and density of surface peptide/MHC class I complexes expressed on murine DCs [17]. In some cases, radiotherapy can lead to upregulation of a class of immunogenic potential tumor rejection antigens, the cancer-testis (CT) antigens in sarcoma patients [18] which can be targeted with adoptive T cell therapy and other antigen-specific immune-based approaches.

One significant hurdle faced by T cell-based immunotherapies is down-regulation of MHC genes which may represent an important mechanism by which tumors evade host immune surveillance, especially among tumor cells breaching the interface between normal and malignant tissues. To test this hypothesis, the tumor cells in mouse GL261 gliomas were treated with radiation therapy, and effectiveness of immunotherapy was examined. GL261 glioma cells showed higher levels of MHC class I molecules not only *in vitro* but also *in vivo* upon radiation and, in combination with vaccination, resulted in significant increases in CD4^+^ and CD8^+^ T cells, and NK cells infiltrating tumor sites. This study demonstrated that the combined radiation and vaccination therapies can restore host immune surveillance in mice through upregulation of MHC class I and produce successful outcomes [19].

In other tumors, high-dose γ-irradiation of human multiple myeloma (MM) cell lines such as ARP-1, ARK-RS, and 10 MM primary tumors, led to upregulation of MHC class I and II molecules in dose-dependent fashion [20].

### 2.3. Immune Modulators; HMGB-1 and TLRs

Some types of tumor cell deaths can induce a DC-mediated cytotoxic T lymphocyte (CTL) response, wherein calreticulin, a Ca^2+^ binding protein, becomes exposed on the cell surface during immunogenic cell death. However, in cases where calreticulin exposure may not be sufficient to elicit the anti-tumor immune response, other proteins such as HMGB1, a soluble protein arising from dying tumor cells, may play an essential role in anti-tumor immunity through its interaction with TLR4 on antigen-presenting cells such as DCs and macrophages. During radiotherapy and chemotherapy, this soluble danger signal is released from dying tumor cells, activates TLR4 signaling through its MyD88 adaptor in DCs and promotes efficient processing and cross presentation of tumor antigens. This immunoadjuvant pathway is believed to be clinically relevant; TLR4 for example has been shown to contribute to the immune response observed in patients whose breast cancers relapse more quickly after chemoradiotherapy [21].

An *in vitro* assay has been developed, as a surrogate indicator of response to potentially immunogenic chemoradiotherapy. Recent studies [22] have shown that about 38% of patients with esophageal squamous cell carcinoma (ESCC) had tumor antigen-specific T cell responses as well as elevated serum HMGB1. This response was up-regulated in patients with ESCC who received preoperative chemoradiotherapy, but not in those patients who did not received chemoradiotherapy; a positive correlation was observed between HMGB1 serum levels and patient survival. Thus, the TLR4-MyD88-HMGB1 pathway in DCs can be manipulated to induce or enhance the CTL-dependent abscopal effect in various tumors.

### 2.4. Regulatory T cells

Regulatory T cells, characterized by the expression of intracellular FoxP3 and a specific surface marker profile (CD25^+^, CD127^−^) serve a physiologic role under normal conditions to suppress an overly vigorous cellular immune response that may otherwise incur serious autoimmunity or bystander immunopathology. In the tumor setting, regulatory T cells may be co-opted by tumor cells to inhibit anti-tumor T cell activity. For full effector function to be realized, strategies to attenuate or eliminate the Treg response would be desirable. In poorly immunogenic tumor models, such as B16 melanoma, spontaneous CD8^+^ T cell-mediated anti-tumor immunity rarely develops; however, deletion of CD4^+^ T cells in B16 tumor-bearing mice uncovers a robust endogenous tumor antigen specific CD8^+^ T cell response capable of inducing tumor regression, manifest as an abscopal effect; furthermore, adoptive transfer of CD8^+^ and CD4^+^ T cells in *Rag1*^−/−^ mice, lacking CD4^+^CD25^+^ compartment elicited the robust concomitant immunity that was attenuated with the addition of CD4^+^CD25^+^ cells [23].

In general, it is believed that regulatory T cells are more radio-resistant than conventional effector T cells [24] and may be over-represented in patients receiving radiation therapy compared with radiation-naïve patients [25], a finding that is recapitulated in animal models [26]. Radiation induced upregulation of TGF-β production, and adenosine A_2A_ in head and neck squamous cell carcinoma (HNSCC) patients [27] can provide both a growth and survival advantage to Tregs [28], thereby suppressing the potential beneficial anti-tumor effects of radiotherapy. Strategies to eliminate this advantage for Tregs by adjusting the dose and schedule of radiation therapy and suppressing the activity or numbers of Tregs when concomitant radiation therapy is administered would be desirable and enable the endogenous anti-tumor response to emerge and its anti-tumor effects enhanced by radiation therapy.

### 2.5. Clinical Implications of Concomitant Immunity

Although the abscopal effect has been observed in both *in vitro* and *in vivo* animal models, this phenomenon has only recently been reported in the clinical setting.

In most cases these responses were observed in patients with lymphoid malignancies wherein, radiation or treatment of local disease led to regression in distant unirradiated sites [29–31]. However, notable cases have been observed in Merkel cell carcinoma [32], advanced uterine cervical carcinoma [33] and hepatocellular carcinoma (HCC). Although radiation to lymphoid sites affected by disease may be more likely to incite systemic immunity due to the higher likelihood of immune effectors trafficking through these regions and encountering released antigen, irradiation to visceral sites of disease including bone, skin and parenchyma was also capable of inducing an abscopal effect [8,33,34].

In cancer therapies, some notions of metastasis and recurrence may be explained using oligometastases and oligo-recurrence. Oligometastases is the state capable of achieving long-term survival or cure with local therapy despite active primary legions. On the other hand, oligo-recurrence is the notion that metastatic and recurrent lesions could be treated with local therapy since the primary lesions have been controlled [35–37]. In oligometastases, stereotactic body radiotherapy (SBRT) provides a treatment option for deep-seated tumors using oligo-fractionated delivery of high-dose radiation while minimizing damage to normal tissues [38]. This high-dose ablative radiation therapy can also be employed in combination strategies such as adoptive cell therapy and anti-CTLA-4 therapy. Administration of autologous DCs, produced *ex vivo* through autologous leukapheresis derived monocytes, can also boost immune responses presumably by facilitating presentation of tumor antigens released during radiation therapy [39]. In clinical trials, the addition of SBRT to high-dose IL-2, has been shown to be highly effective in patients with metastatic melanoma and renal cell cancer and represents a clinically tenable strategy given that HD IL-2 is approved for use in these malignancies. The presence of an elevated effector memory CD4^+^ T cell population in the peripheral blood was associated with a clinical response in these patients [40].

## 3. Effects of Radiation on Transferred Immune System

**Figure 1.**
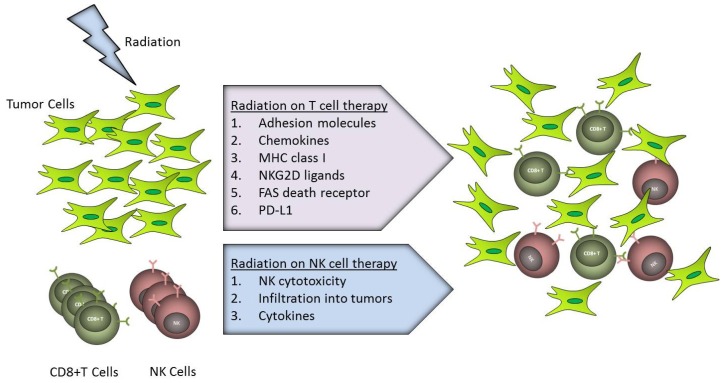
Radiation enhanced T and Natural Killer (NK) cell therapies against tumors. Radiation up-regulates adhesion molecules such as ICAM-1 and E-selectin on tumor cells as well as chemokines in tumor microenvironment, helping immune cells trafficking. More effector CD8^+^ T cells infiltrate into tumors due to higher expression of MHC class I, NKG2D ligands, FAS or PD-L1 on target cells upon irradiation. In case of NK cells, radiation increases NK cells cytotoxicity against tumors as well as lymphocytes trafficking into tumors and cytokines production.

As described above, the endogenous immune system can be modulated to mount an effective anti-tumor response following radiation therapy and, in combination with other immunomodulators, this effect can be further potentiated. However, such approaches are constrained by the relatively low extant frequency of tumor-reactive immune cells (T cells and NK cells), and presence of suppressive factors (regulatory T cells myeloid derived suppressor cells, metabolic inhibitors) limiting *in vivo* immune cell expansion and activation. Adoptive cellular therapy involving the *ex vivo* enrichment, isolation and expansion of tumor-reactive T cells and NK cells can circumvent some of these obstacles associated with induction of an afferent immune response and, while they may be subject to some of the same suppressive factors *in vivo*, adoptive therapy allows greater control to be exerted over the magnitude, phenotype and specificity of the intended immune response. Adoptive T and NK cell therapies are discussed below in the context of combination with radiation therapy, as briefly depicted in Figure 1.

### 3.1. Adoptive T Cell Therapy

Adoptive cellular therapy involves the *ex vivo* isolation and expansion of tumor-reactive T cells for infusion with the expectation that these T cells will traffic to tumor sites, eradicate tumor and provide long term immunoprotection. Adoptively transferred cells can be described as (1) tumor-infiltrating lymphocytes, derived from a tumor biopsy which has been disaggregated and cultured in the presence of high-dose IL-2 to enrich for a population of tumor-reactive T cells from the mixed tumor-T cell population; (2) chimeric antibody receptor (CAR) or T cell receptor-engineered lymphocytes generated by transfection of a vector encoding the antibody or T cell receptor recognizing the ligand of interest; (3) antigen-specific T cells present in very low frequency in the peripheral blood, selected and enriched using specialized *in vitro* culture approaches. Although each of these approaches has demonstrated some very dramatic durable complete responses, a significant fraction of patients fail to respond to adoptive T cell therapy. In addition to designing approaches to augment the *in vivo* persistence of transferred T cells, strategies to improve the capacity of T cells to infiltrate tumor sites and oppose negative influences within the tumor microenvironment, both features of which can be addressed by concomitant radiation therapy, would lead to enhanced immune responses and clinical outcomes. Concomitant administration of radiation therapy can benefit adoptive T cell therapy by facilitating tumor trafficking and upregulating molecules on tumor cells that facilitate recognition and killing.

#### 3.1.1. Lymphocyte Trafficking

Murine models of adoptive transfer have examined if radiation can enhance the infiltration of T cells into solid tumors [41,42]. In one study, radiation of subcutaneously inoculated B16-OVA at tumor sites led to increased priming of tumor-reactive T cells in the draining lymph node and accumulation of endogenous tumor antigen-specific CD8^+^ and CD4^+^ T cells infiltrating the tumor; adoptive transfer of *ex vivo* activated OVA-specific OT-1 CD8^+^ T cells led to increased infiltration of transferred T cells to tumor sites (and not merely expansion of localized T cells) following local radiation and was particularly evident with single dose (15 Gy), compared with fractionated dose deliver [41]. This effect was antigen-specific as transgenic T cells recognizing an irrelevant antigen did not infiltrate B16-OVA tumor sites. In a second model using Rip1-Tag2 mice, whereby the SV40 T antigen is expressed by the insulin-promoter, spontaneous pancreatic islet cell tumors developed in the context of systemic tolerance to the tumor-associated T antigen. Adoptive transfer of T antigen-specific CD4^+^ T cells following sublethal irradiation was more effective in controlling tumor burden and extending survival of tumor-bearing mice than with either modality alone. Increased tumor infiltration with transferred Tag-specific CD4^+^ T cells was also observed in irradiated mice [42].

Several mechanisms mediating lymphocyte infiltration into tumors have been advanced. One potential mechanism involves triggering of inflammation. Localized irradiation of the tumor site can modify the microenvironments generating inflammatory cytokines, thereby increasing trafficking and retention of T lymphocytes within tumors. IFN-γ is one such proinflammatory cytokine with important roles in immune responses to tumors, including modulation of tumor-specific CTL effector functions, and inhibition of tumor cell proliferation and angiogenesis. Some IFN-γ-mediated anti-tumor effects are mediated through caspase activation, surface MHC class I expression and up-regulation of IFN-γ inducible genes such as antiangiogenic chemokines within tumor cells. To determine how IFN-γ influences the inflammatory responses within this dynamic environment following radiotherapy, B16/OVA melanoma cells were implanted into wild-type C57BL/6 and IFN-γ deficient mice. Expression of VCAM-1 and MHC class I was up-regulated on the tumor vasculature of WT but not *Ifn-γ*^−/−^ mice. In comparison, levels of IFN-γ-inducible chemokines like MIG (an antiangiogenic chemokine) and IFN-γ-inducible protein 10 were decreased in irradiated tumors from *Ifn-γ*^−/−^ mice. In particular, IFN-γ acts directly on tumor cells in mice to up-regulate MHC class I, whose higher expression is correlated with greater STAT1 activation, leading to a tumor microenvironment conducive for T cell infiltration and tumor cell target recognition [43]. ICAM-1 has also shown to be upregulated in human multiple myeloma cell lines and primary tumor cells following exposure to high-doses of irradiation and facilitates T cell infiltration into tumors and itself, may be presented as an antigenic protein on the surface of tumor cells [20]. The antigenic expression of ICAM-1 elicited at gene expression level has also been observed in human colonic BM314 and gastric MKN45 adenocarcinoma cells after ionizing irradiation [44].

Another mechanism of radiation-induced lymphocyte infiltration into tumor microenvironment involves up-regulation of chemoattractants MIG and IP-10. These chemoattractants appear to promote IFN-γ responses by conditioning the tumor microenvironment for enhanced CTL trafficking and recognition of tumor cells in the context of radiation [43]. Chemokines and their cognate receptors modulate the migration of effector T cells to different inflamed tissues in stimulus- and organ-dependent ways. Ionizing radiation therapy significantly enhanced the secretion of CXCL16 in mouse and human breast cancer cells. Since Th1 and CD8^+^ effector T cells express the CXCL16 counterreceptor CXCR6, radiation therapy has been shown to markedly enhance the migration of CD8^+^ CXCR6^+^ activated T cells to tumors. Thus, the same proinflammatory chemotactic factor recruits antitumor effector CD8^+^ T cells into tumors, while converting tumors into inflamed peripheral tissues [45]. Other chemokines such as CCL2 have also been shown to play a role in tumor homing of immune cells in mice following radiation [46].

#### 3.1.2. Tumor Recognition and Killing

Cytotoxic CD8^+^ T cells are an essential component of immune based therapies whose effector function is dependent on MHC Class I presentation of tumor-derived epitopes. One mechanism by which radiation may potentiate CTL mediated tumor control involves upregulation of MHC class I molecules on tumor or antigen presenting cells thus facilitating direct antigen presentation and cross presentation to CD8^+^ T cells. Low-dose radiation can upregulate MHC class I expression to high levels on glioma cells in mice leading immune-mediated tumor eradication *in vivo* [19]. By comparison, other tumor types (e.g., multiple myeloma) require high-dose γ-irradiation to achieve enhanced expression of MHC class I/MHC class II, increased immunogenicity and improved efficacy when combined with immunotherapy [20].

Upregulation of costimulatory ligands such as that for NKG2D (an activating receptor expressed on CD8^+^ T cells and NK cells) can potentiate anti-tumor cytotoxicity. NKG2D ligands are upregulated on murine tumor cells following stress-inducing events such as exposure to DNA damaging agents like high-dose of ionizing radiation and inhibitors of DNA replication such as mitomycin C, hydroxyurea, 5-fluorouracil (5-FU) [47].

Another pathway associated with tumor recognition and killing that is influenced by radiation exposure is the Fas-dependent apoptosis pathway. Elevated levels of the fas death receptor on cancer cells can enhance the efficiency of CTL-mediated killing. In the human CEA transgenic mouse model, irradiation on tumor cells up-regulated Fas expression and led to the sensitization of tumor cells to adoptively transferred antigen specific CTL [48]. Based on the hypothesis that low doses of radiation may engender enhanced susceptibility of cancer cells to cytotoxic CD8^+^ T cell immunity, 23 human carcinoma cell lines (12 colon, 7 lung, and 4 prostate) were examined for their responses to nonlytic doses of radiation [49]. Twenty-one of 23 cancer cell lines (91%) up-regulated expression of one or more of surface molecules such as Fas (CD95), ICAM I and MHC class I associated with T cell effector function. This study suggests that nonlethal doses of radiation can render human tumors more amenable to immune system recognition and attack, providing a rationale for the combined use of adoptive cellular therapy and local tumor irradiation. A single dose of radiation to tumor can induce up-regulation of death receptor Fas *in situ* for up to 11 days. However, only the combined therapy of radiation at this dose and vaccines was able to cure the established tumors which neither of these therapies alone couldn’t treat. This combinatorial therapy demonstrated a massive T cells infiltration specific for tumor antigens in mice [50].

One caveat with the use of radiation therapy is that upregulation of the inhibitory receptor ligand, PD-L1, can be induced on tumor cells. Optimization of combined radiation and T cell-based therapy can be achieved using a PD-L1 blocking antibody and in animal models, radiation-induced CD8^+^ T cell immunity could be rescued from the PD-1/PD-L1 inhibitory signaling pathway using anti-PD-L1 led to dramatic increase in the antigen-specific T cell pool in the draining lymph nodes and complete tumor eradication in mice treated with the combination of anti-PD-L1 blockade and radiation therapy [51].

Taken together, several lines of investigation indicate that radiation boosts tumor-specific immune responses via multiple pathways, making these pathways a novel therapeutic target to enhance immunogenicity of tumor cells while reducing overall toxicity.

### 3.2. Adoptive Natural Killer (NK) Cell Therapy

NK cells provide a body’s the first line of defense against tumor cells and function without the requirement for prolonged pre-activation [52,53]. In contrast to antigen-specific T cell therapy, identification of the target tumor antigen is not required for NK cell therapy which can be more universally applied and particularly effective for treating solid tumor malignancies that have lost expression of self-MHC as a mechanism of immune escape from effector T cells [54,55].

Although Natural Killer (NK) cells are more radio-resistant than T and B lymphocytes in rats [56], they are still sensitive to high-dose of radiation that abrogates their cytotoxicity against tumor cells. Despite intact binding to tumor cell targets following irradiation, NK cells failed to get activated after conjugate formation and thereby incapable of degranulation. Interestingly, culturing NK cells with IL-2 prevented them from losing anti-tumor cytotoxic functions under irradiation [57]. In contrast, low-dose of radiation alone augmented natural cytotoxicity of NK cells against tumor targets cells. Even NK cell-resistant T24 bladder transitional carcinoma cells showed increased sensitivity to killing by blood lymphocytes along with X-ray irradiation [58,59]. The radiation-mediated increase in NK cytotoxicity seems to be peaking at an optimal dose and declining after the peak [60]. It was shown that the increase of NK cytotoxicity could also be maintained long term via co-culturing with interferon-α immediately after irradiation [59]. The augmented NK cell cytotoxicity was induced without any phenotypic changes in NK cells, e.g., NK1.1, NKG2D, CD69 and 2B4 expression, or changes in the rate of early or late apoptosis [61,62]. The results from *in vitro* studies were also confirmed in an *in vivo* mouse model, demonstrating that mice exposed to low-dose radiation exhibited stimulation of innate immunity while suppressing pro-inflammatory responses [62]. Furthermore, low dose irradiation enhanced the cytotoxic effects of NK cells against tumor cells *in vivo*, when NK cells were inoculated as a mixture with tumor cells into mice after irradiation [63]. Together, these results demonstrate that low-dose radiation can modulate NK cell sensitivity against tumor cells, leading to increased tumor killing.

The differential anti-tumor responses might be expected from various mouse strains, since BALB/c and C57BL/6 mice seem to differ in their Th1/Th2 lymphocyte and M1/M2 macrophage phenotypes [64]. In terms of radiosensitivity, however, repeated exposures of 2 mouse strains to low level X-rays resulted in comparable upregulation of NK cell cytotoxicity as well as similar levels of increases in anti-tumor activities of macrophages. Thus, the similar anti-tumor responses in these mouse strains after irradiation suggested very little variation among strains, not involving distinct cytokine signatures displayed in each mouse, in radiation-mediated anti-tumor immunity [64].

To understand the mechanisms by which NK cells acquire enhanced anti-tumor activity with irradiation, various cancer lines were co-cultured with human NK cells. NK cells exhibited time- and dose-dependent enhancement in their anti-tumor cytotoxicity with irradiation, whose primary mechanism was the caspase activation via perforin/granzyme B after cell-cell contact. Radiation appeared to induce Smac release from mitochondria and neutralize XIAP and thereby increase NK cell killing [65]. Short wavelength ultraviolet light irradiation on MCA 102 tumor cells established a new cell line MCA 102 UV, which has increased immunogenicity and higher sensitivity to killing both by natural killer (NK) cells and natural cytotoxicity (NC) cells in normal spleen cells, while the latter ones mostly mediate the lysis of normal MCA 102 cancer cells [66]. Previous studies indicated that UV irradiation increased tumor cell susceptibility to NK cell-derived lytic granules without any effects on tumor cell recognition by NK cells [67]. On the other hand, the enhancement of UV-treated tumor cell sensitivity to lysis by NC cells was due to their increased sensitivity to TNF-α released from NK cells [66–68].

Interestingly, when INF-γ and endostatin transfected tumor cells were irradiated *in vivo* in murine model, tumor cell growth and metastases were alleviated through IFN-γ-stimulated CTL and NK cell activation and endostatin-induced antiangiogenic activity [69]. Although NK cell activity and their numbers inside tumors have positive correlations with improved prognosis for cancer patients, application of NK cells to immunotherapy has had limited successes due to their short-lived effector functions. Therefore, infiltration of NK cells into tumors and their effective and long-lived efficacy *in vivo* have been intensively investigated for many years. Recent studies demonstrated that murine NK cells could be preactivated with IL-12, IL-15 and IL-18 *in vitro* and have potent anti-tumor activity against tumors. The IL-12/1L-15/1L-18-preactivated NK cells showed greatly higher numbers and persistent effector functions inside established murine tumors with radiation therapy, highlighting the therapeutic modalities of combined NK cell and radiation therapy [70]. Aside from the preactivation of NK cells for enhanced *in vivo* efficacy, the acquisition of enough numbers of NK cells *in vitro* is another concern in NK cell therapy. This *ex vivo* expansion could also be accomplished *in vitro* using autologous/allogeneic feeder cell, irradiated lymphocytic leukemia cells, KL-1 cells [71] and gene-modified K562 monocytic leukemia cells [72,73]. Therefore, low dose radiation would be beneficial in activating anti-tumor cytotoxicity and cytokine production in both endogenous NK cells as well as adoptively transferred NK cells.

## 4. Conclusions

In development of novel therapies for the treatment of patient with cancer, the use of radiation alone can produce significant local control and in recent studies, has also been shown to mediate anti-tumor responses at distant sites by triggering and enhancing the endogenous cellular immune responses. This process can involve multiple pathways leading to increased antigen availability, antigen presentation, tumor sensitization and immune cell trafficking. As the dose and schedule of delivering radiation to the host can alter the tumor microenvironment, an understanding of these mechanisms can be explored in preclinical studies and applied to the clinical arena to improve patient outcomes. With the increased availability of immunomodulatory reagents and the development of novel strategies to manipulate the antigen-specific immune response by vaccination, immunomodulation and, more recently, adoptive cell transfer, the combination of radiation therapy and one or more immune-based modalities represent a potent synergistic approach to providing long term protection and minimal toxicity.
